# Structure, Oligomerization, and Thermal Stability of a Recently Discovered Old Yellow Enzyme

**DOI:** 10.1002/prot.26800

**Published:** 2025-01-22

**Authors:** Nakia Polidori, Peter Babin, Bastian Daniel, Karl Gruber

**Affiliations:** ^1^ Institute of Molecular Biosciences University of Graz Graz Austria; ^2^ Dipartimento di Scienze della Vita e Biologia dei Sistemi Università di Torino Torino Italy

**Keywords:** ene‐reductase, old Yellow enzymes, oligomerization, oxidoreductase, thermophilic enzymes, thermostability

## Abstract

The Old Yellow Enzyme from *Ferrovum sp. JA12* (FOYE) displays an unusual thermal stability for an enzyme isolated from a mesophilic organism. We determined the crystal structure of this enzyme and performed bioinformatic characterization to get insights into its thermal stability. The enzyme displays a tetrameric quaternary structure; however, unlike the other tetrameric homologs, it clusters in a separate phylogenetic group and possesses unique interactions that stabilize this oligomeric state. The thermal stability of this enzyme is mainly due to an unusually high number of intramolecular hydrogen bonds. Finally, this study provides a general analysis of the forces driving the oligomerization in Old Yellow Enzymes.

## 1 Introduction

**SCHEME 1:**
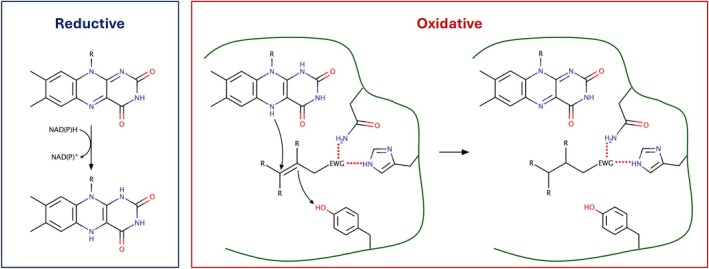
General reaction mechanism of Old Yellow Enzymes, divided into reductive and oxidative half‐reactions.

Old Yellow Enzymes (OYEs) are a broad family of flavoproteins with a modified TIM‐Barrel fold, adapted for the binding of the 5′‐phosphate of the FMN. These enzymes catalyze the reduction of a broad range of unsaturated carbonyl [1, 2, 3, 4, 5, 6] and nitrocompounds [7, 8, 9], creating stereocenters with high degrees of stereoselectivity. The reaction occurs through a ping‐pong mechanism, with a reductive and an oxidative half‐reaction [10]. During the reductive half‐reaction, the enzyme binds NAD(P)H, and its cofactor is reduced by this molecule through hydride (H^−^) transfer. Subsequently, the NAD(P)^+^ is released, and the oxidative half‐reaction can begin. The substrate diffuses into the active site and here interacts with two polar residues, either a histidine–asparagine or a histidine–histidine couple. The hydrogen bonds of these residues with the electron‐withdrawing group near the double bond activate the substrate for the reaction. The substrate accepts two electrons from the reduced cofactor (FMNH_2_) in the form of a hydride; the consequent negative charge is readily neutralized by a proton transfer, which generally occurs from a highly conserved tyrosine residue (Scheme 1).

**FIGURE 1:**
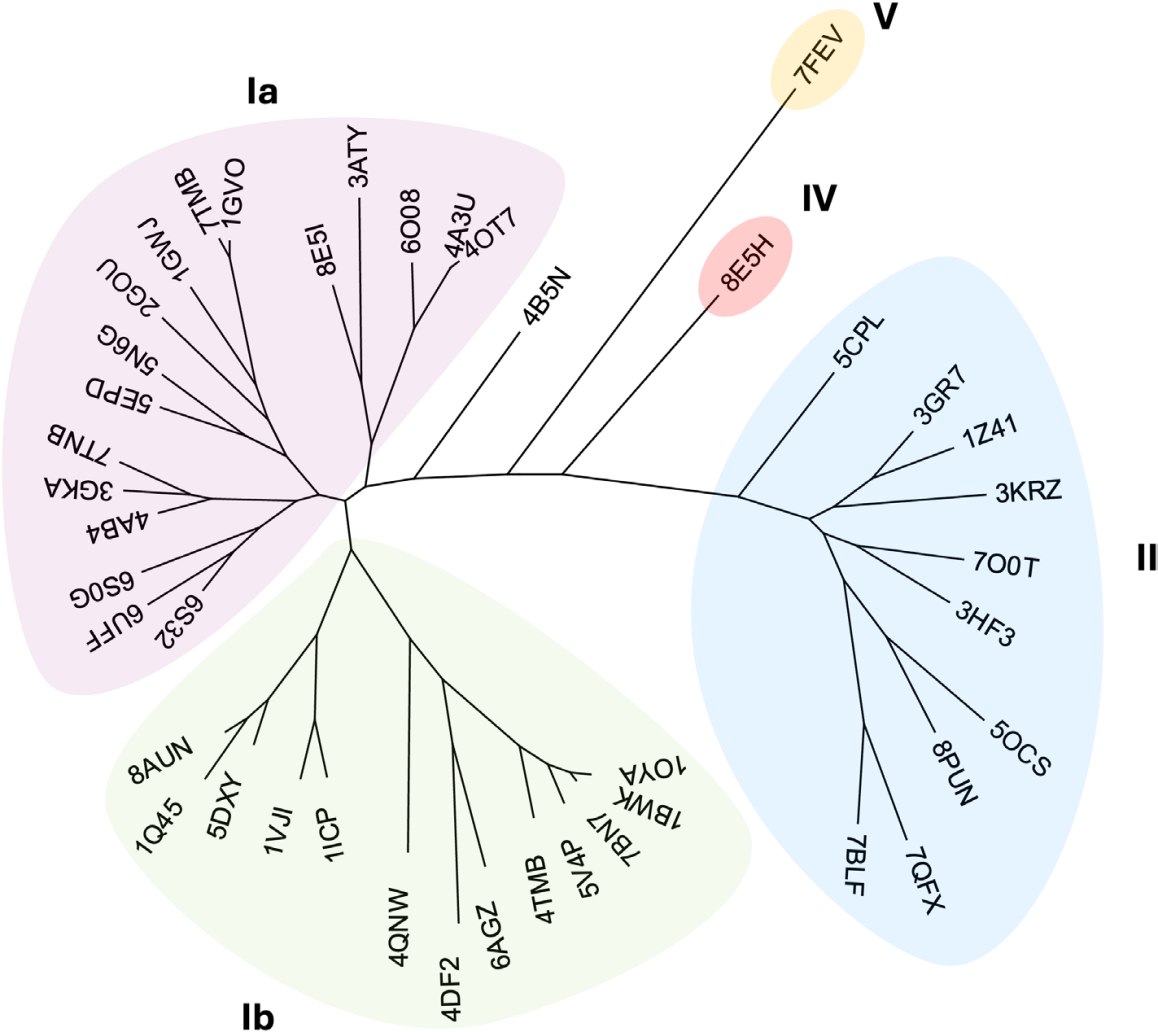
Unrooted tree of OYE structures present on the Protein Data Bank. Classification is based on the work of Peters et al. [12]. FOYE has a tetrameric structure.

Because of their potential as biocatalysts [11], OYEs constitute one of the most characterized families of enzymes. To date, more than 100 OYE structures have been deposited on the Protein Data Bank, with 43 unique enzymes assembled as monomers, dimers, and tetramers. Recent classifications divide OYEs into five classes [12]: Class I, also known as classical OYEs, can be divided into bacterial (Ia) [13, 14] and fungal (Ib) enzymes [15]; Class II, also known as thermophilic‐like [16, 17]; Class III and Class IV, both very little characterized [12, 18, 19], though the structure of a Class IV member was recently determined (PDB: 8E5H); and Class V, with the recently crystallized ArOYE6 [20]. A phylogenetic tree containing unique structures deposited on the PDB is presented in Figure 1.

In 2017, Scholtissek et al. [21] isolated a new OYE from the acidophilic iron oxidizer *Ferrovum* sp. JA12. This enzyme (FOYE), belonging to Class II OYEs, was thoroughly characterized, and despite coming from a mesophilic bacterium, it displayed an unusual thermal stability. Subsequently, Velikogne et al. [22] used this enzyme to catalyze the conversion of α‐oximo β‐keto esters to the related amines. We determined the crystal structure of this enzyme and found that it displays a tetrameric structure similar to other OYEs. After analyzing the residues that participate in this particular oligomerization, we further investigated the thermal stability of this enzyme through CD spectroscopy and bioinformatic analyses.

## 2 Materials and Methods

### 2.1 Vector and Construct Design

The construct for protein expression was kindly donated by Wolfgang Kroutil (University of Graz). The gene‐encoding FOYE (WP_056929840) was cloned in the pET‐28a(+) vector using the restriction sites NdeI (5′) and XhoI (3′). The protein expressed in this construct bears an N‐terminal 6xHis tag combined with a thrombin cleavage site. The DNA and protein sequences are available in the Supporting Information. The construct was cloned in competent *Escherichia coli* BL21(DE3) Star (pLysS) cells using a standard heat‐shock protocol. The transformed colonies were selected on agar plates containing 40 μg/mL kanamycin for vector selection and 34 μg/mL chloramphenicol for strain selection (pLysS).

### 2.2 FOYE Gene Expression and Enzyme Purification

Precultures containing LB medium, antibiotics, and an aliquot of the glycerol stock were incubated overnight to prepare the main culture inoculum. The main culture was inoculated with 1% v/v preculture, and cultures were grown on a shaker at 37°C until they reached an OD_600_ of 0.6–0.8. Gene expression was induced at this point with 0.5 mM IPTG, and the cultures were incubated overnight at 25°C.

The cultures were harvested by centrifugation, and the cells were disrupted by sonication after being resuspended in lysis buffer (100 mM TRIS, 300 mM NaCl, 25 mM imidazole, pH 8.0). The supernatant was separated from the cell debris by ultracentrifugation, and the protein was purified by IMAC. Briefly, the supernatant was loaded on a HisTrap FF 5 mL column (Cytiva) using an Äkta Pure system (Cytiva). The immobilized protein was washed with 10 column volumes of washing buffer (100 mM TRIS, 300 mM NaCl, 25 mM imidazole, pH 8.0) and subsequently eluted *via* isocratic elution with the elution buffer (100 mM TRIS, 300 mM NaCl, 300 mM imidazole, pH 8.0). The eluted fractions containing the protein of interest were concentrated, and the enzyme was further purified *via* size‐exclusion chromatography (SEC) on a Hiload 16/60 Superdex 200 column. The pure enzyme was eluted using SEC buffer (20 mM TRIS, 100 mM NaCl, pH 8.0). The pH of all buffers was adjusted using HCl.

### 2.3 Protein Crystallization and Data Collection

The purified protein was concentrated to 5–15 mg/mL, and crystallization trials were set up using the sitting‐drop vapor diffusion method. The plates were incubated at 20°C, and yellow, needle‐shaped protein crystals appeared after approximately 5 days in several conditions from different commercial screens. Three‐dimensional, well‐diffracting crystals were obtained in condition E4 of Morpheus II screen using a protein concentration of 10.9 mg/mL. Any attempt at optimization failed to improve the quality of the crystals.

Diffraction data were collected at ESRF Beamline ID30B (Grenoble). Diffraction images were processed using the AutoPROC [23] pipeline, including XDS [24], Pointless [25], Aimless [26], CCP4 [27], and STARANISO (Global Phasing Ltd., United Kingdom). The structure of FOYE was solved by molecular replacement using Phaser, available in the Phenix package [28], and a homology model was generated using SwissMODEL [29] as a search template. The structure of the undeposited molecule trimethylolpropane was generated using eLBOW available in the Phenix package [28], giving the Simplified Molecular‐Input Line‐Entry (SMILE) sequence as input. Ligands were added to the structure using Coot [30], and the refinement was carried out using Phenix refine [28]. Statistics of X‐ray data collection and refinement are reported in Table S1. The structure is deposited in the Protein Data Bank (PDB) under the accession code 8PUN.

### 2.4 Analytical Size‐Exclusion Chromatography

Gene expression and protein purification *via* IMAC were conducted as described before.

The fractions containing FOYE were pooled and concentrated to approximately 10 mg/mL. Five‐hundred microliters of protein sample was loaded on a Superdex Increase 10/300 GL size‐exclusion chromatography column (Cytiva) mounted on an Äkta Pure system (Cytiva). Fractions were eluted with a flow rate of 0.2 mL/min using SEC buffer (20 mM TRIS, 100 mM NaCl, pH 8.0) and collected for SDS‐PAGE analysis. The calibration curve for the elution volumes was built using the gel filtration standard (Bio‐Rad), with the same flow rate and buffer. The elution profiles, elution volumes, and calibration curve are represented in Figure S2.

### 2.5 Phylogenetic Tree Construction

The sequences of Old Yellow Enzymes with available structures were found using a BlastP search on the Protein Data Bank, using the sequence of FOYE as the query. After removing all the duplicates and amino acid variants, the remaining protein sequences were inspected, and eventual purification tags were removed before the alignment.

For Class II OYE tree, Class I GsOYE was used as an outlier. The maximum likelihood distance trees and the corresponding alignments produced *via* Clustal W were generated using MEGA11 software [31].

### 2.6 Hydrogen Bonds, Salt Bridges, and Proline Content

The analysis for intra‐ and intermolecular polar interactions was done using the Hbplus software v3.2, available on LigPlot+ [32]. The polar contacts were calculated separately for monomers, dimers, and tetramers to account for the interactions established in the oligomerization process. The proline content was calculated by counting the proline residues in each sequence and dividing it by the total number of residues.

### 2.7 CD Spectroscopy and Melting Temperature

For CD measurements, the SEC purified protein was used upon buffer exchange to 50 mM KPi, pH = 7.5. The CD measurements were performed on a Jasco J‐1500 CD spectrometer. The protein was diluted to 0.15 mg/mL in order to obtain a signal between 10 and 20 mdeg. For measuring the melting temperature, two wavelengths (216 and 222 nm) were chosen for comparison. A single upscan was performed, increasing the temperature from 20°C to 95°C. The melting points were determined by Jasco Spectra Manager software for both wavelengths. Melting temperature spectra are available in the Supporting Information.

## 3 Results

### 3.1 FOYE Has a Tetrameric Structure

**FIGURE 2:**
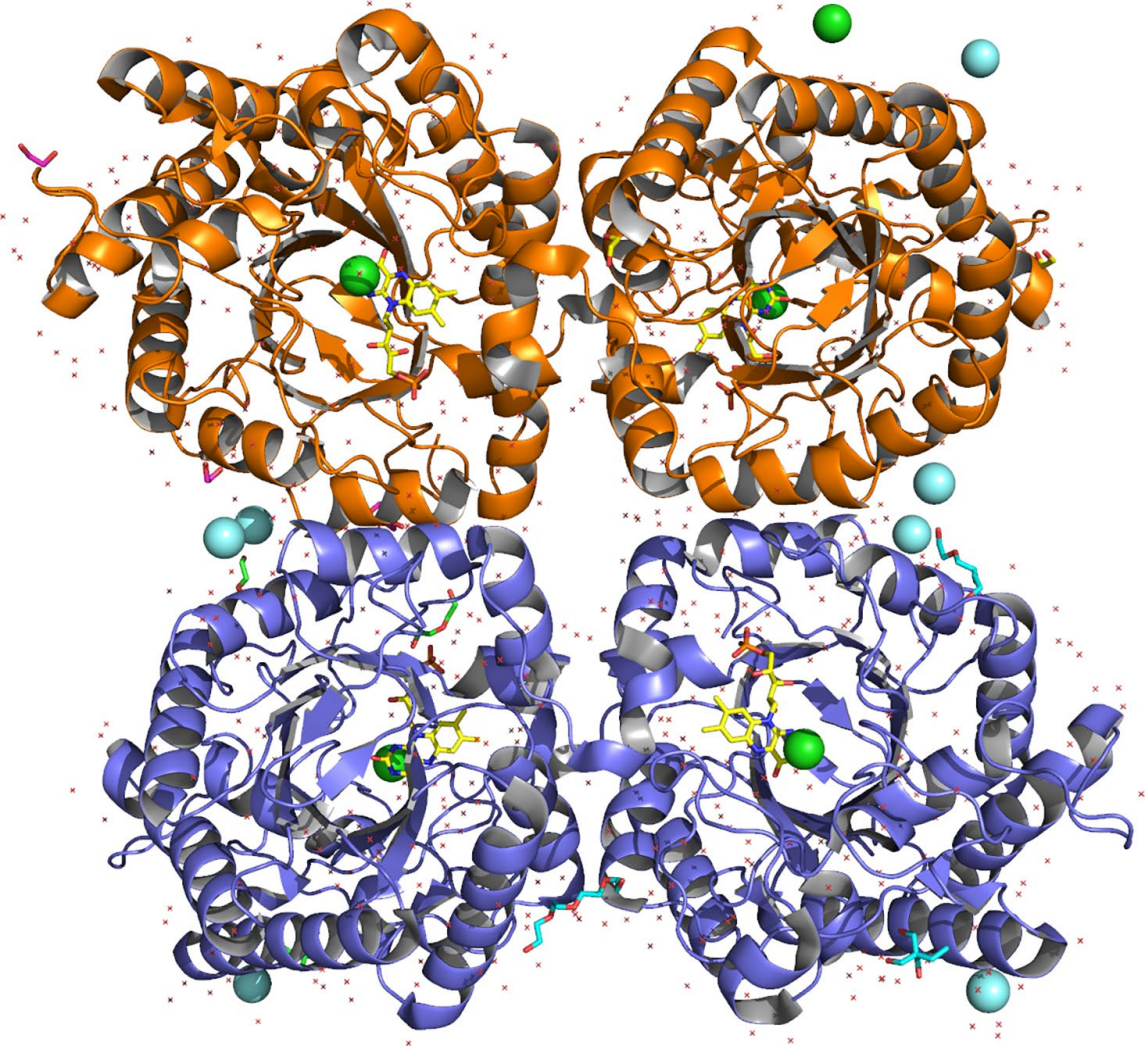
FOYE tetramer, with the two functional dimers highlighted in orange and slate blue.

FOYE exhibits a tetrameric quaternary structure in the crystal with four molecules in the asymmetric unit (Figure 2). The protein elutes as a single peak during SEC (Figure S1), suggesting that oligomerization is not concentration‐dependent. To test if the oligomerization is an artifact of the crystallization conditions, we performed analytical SEC using a column with higher resolution. FOYE elutes again as a single peak, with an apparent molecular weight of 130 kDa (Figure S2). The molecular weight of our construct is 40.7 kDa; thus, the eluted protein has a size larger than a trimer. The EBI‐PISA server also predicts FOYE to be tetrameric based on the crystal structure (Table S2). Additionally, most of the residues and interactions at the tetramerization interface are conserved among the other tetrameric OYEs (see below). Based on this evidence, it is safe to assume that FOYE is also tetrameric in solution.

The four chains display a clear electron density for all the protein residues, plus two extra amino acids from the N‐terminal expression tag. The electron density for the FMN is clearly visible in all the protomers, indicating good retention of the cofactor throughout the purification process. A positive electron density with an unclear shape is present in front of all the FMN molecules. Similar to Fox and Karplus in their structural study of OYE1 [15], we decided to place chloride ions (depicted in green) inside these densities. Other large undefined electron density blobs are visible on the external surface of the enzyme in proximity to negatively charged residues (E150, D151, E153, E283, and E315). Since the reservoir solution contained a mix of lanthanides, we decided to place yttrium ions (depicted in cyan) at these positions.

**FIGURE 3:**
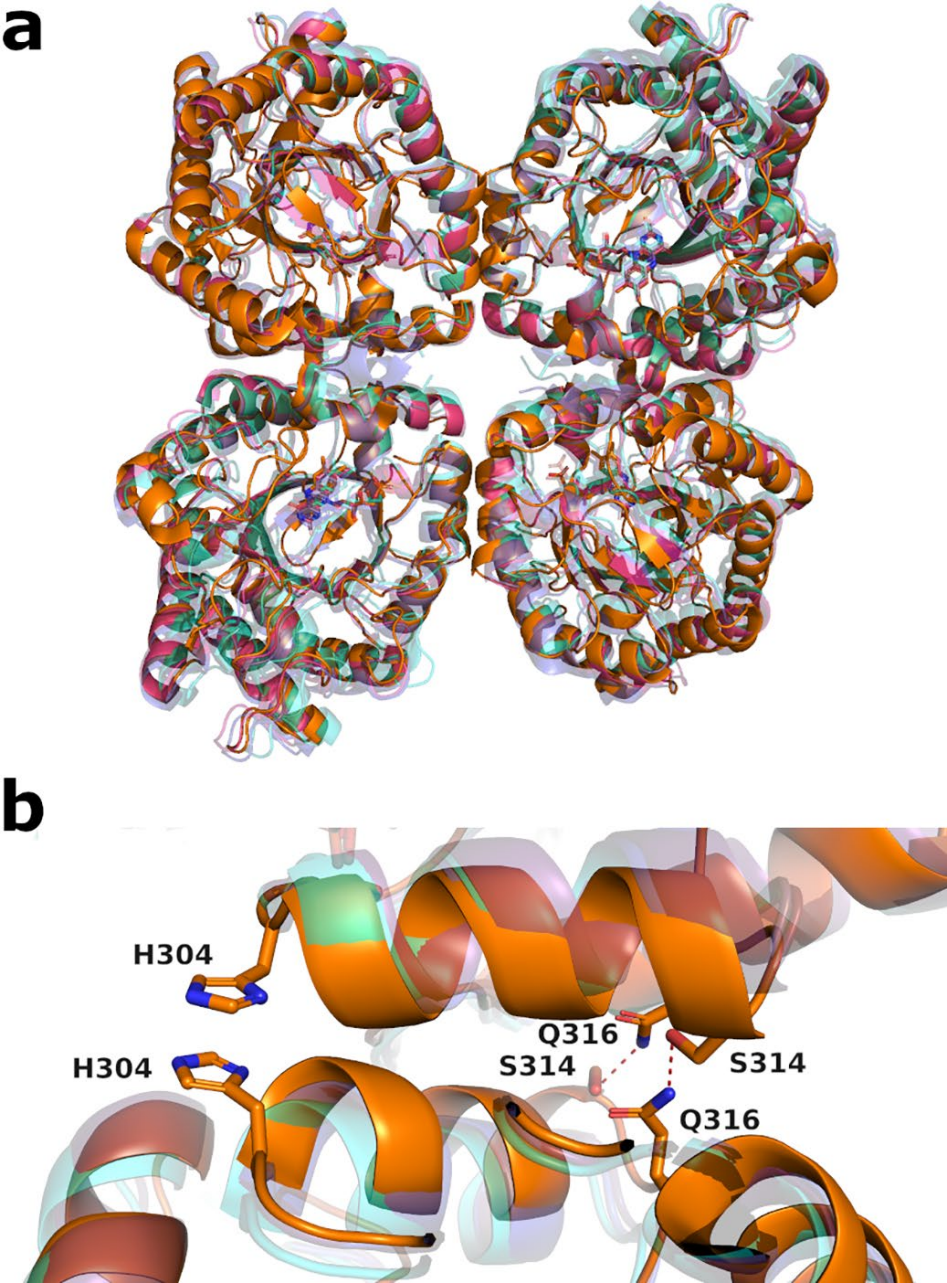
(a) FOYE tetramer (orange) compared to the ones from TOYE (blue), GkOYE (cyan), and Yqjm (purple). (b) Detail on the distortion of helix 7, promoting the tetramerization in FOYE.

The four protomers are virtually identical, and, as for the other tetrameric OYEs [33, 34, 35], FOYE can be considered as a dimer of functional dimers (AB and CD, depicted in orange and lilac, respectively). Curiously, the functional dimers display a higher RMSD (0.177 Å) than the dimers AC and BD (0.058 Å), which share the tetramerization interface. Using the topological map of Yqjm as a reference [34], the tetramerization interface arises from the barrel helixes H6 and H7 and part of the loop L6. The FOYE tetramer is clearly superimposable to the ones of Yqjm, GkOYE, and TOYE (Figure 3a). A small distortion of the helix H7 reduces the distance from the respective counterpart of the other protomer, allowing the formation of a π–π stacking interaction between the histidine residues H304 and a hydrogen bond between S314 and Q316 (Figure 3b).

Despite FOYE clustering separately from Yqjm, GkOYE, and TOYE (Figure S3)—with the highest identity being 45.33%—it preserves the overall spatial location of the interchain polar contacts. For example, N259, which in GkOYE and Yqjm forms a hydrogen bond with Q297, is replaced by the interaction between the backbone of P275 and the side chain of K313 in FOYE. In the same manner, the salt bridge between E294 and R300 in TOYE is substituted by a hydrogen bond between the residues S314 and Q316 in FOYE, which also occupy a similar position as the Y261–N298 pair in GkOYE and TOYE. Notably, FOYE has a unique Q286–Q286 interchain hydrogen bond; in the other enzymes, these residues correspond to E270 and R300, which are in the range to form a salt bridge; however, in the crystal structures of TOYE and GkOYE, they appear with alternative conformations or form intramolecular salt‐bridges. Kitzing et al. [34] reported that hydrophobic interactions greatly contribute to tetramerization in Yqjm. Indeed, most of the hydrophobic interactions are conserved in FOYE and the other tetrameric OYEs, either by maintaining the same residue or by conservative substitutions (Table 1).

**TABLE 1:** Hydrophobic residues conserved at the tetramerization interface. Non‐conservative substitutions are crossed‐out.

Yqjm	FOYE	GkOYE	TOYE
V260	V276	V260	L260
F261	G277	Y261	Y261
P262	P278	P262	P262
Y264	Y280	Y264	Y264
V266	V282	V266	V266
M285	L301	L285	L285
M291			L291

**FIGURE 4:**
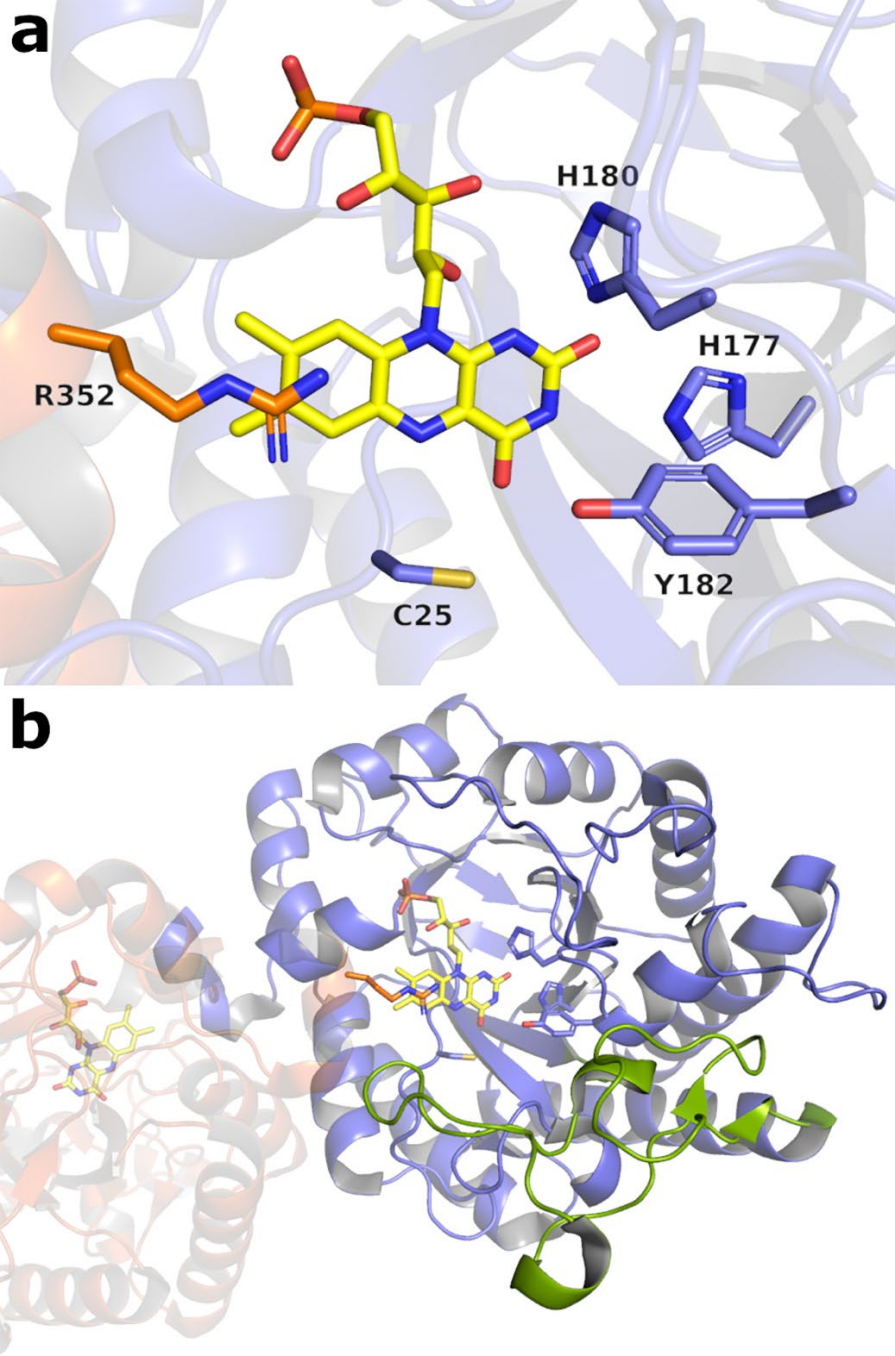
(a) Active site of FOYE, with the conserved histidine residues (H177, H180) and the catalytic tyrosine (Y182) showed as sticks. The typical Class II cysteine residue (C25) is displayed as well. FMN is displayed in yellow. The C‐terminal from the other protomer, with the typical arginine finger (R352) is shown in orange. (b) Unstructured insertion in loop L3 (light green). FOYE thermal stability depends on an elevated number of hydrogen bonds.

The structure of FOYE is also very similar to the ones of the other Class II OYEs like RmER (RMSD 0.725), XenA (RMSD 1.99), and TsOYE (RMSD 2.25). The enzyme displays the signature C‐terminal α‐helix at the dimerization interface, which bears the typical arginine finger (R352) forming part of the other protomer's active site pocket. Moreover, as in the other Class II members, the threonine residue that interacts with the N5 and O4 of the FMN in Class I OYEs is substituted by a cysteine residue (C25 in Figure 4a).

Differently from the other tetrameric OYEs, FOYE displays an extended unstructured region in loop L3 (residues 102–149), which is instead found in the other Class II enzymes, although with no homogenous length (Figure 4b).

### 3.2 FOYE's Thermal Stability Depends on an Elevated Number of Hydrogen Bonds

Regardless of the characterization of FOYE's activity at different temperatures, the melting point of this enzyme was unreported. Consequently, we decided to measure the melting temperature of FOYE using CD spectroscopy (Figure S4). Despite coming from a mesophilic microorganism [36], FOYE displays outstanding thermal stability and a surprisingly high melting temperature (Table 2). Intrigued by this unusual thermal behavior, we tried to unravel the characteristics giving rise to the temperature tolerance of this enzyme.

**TABLE 2:** Melting temperatures of tetrameric OYEs and RmER, the closest homolog to FOYE.

Enzyme (PDB)	*T*_m_	Measuring technique	Thermal behavior	Reference
TOYE (3KRZ)	70°C–79°C	DSF‐DSC	Thermophilic	[33]
GkOYE (3GR7)	81°C	CD	Thermophilic	[35]
Yqjm (1Z41)	51°C	CD	Mesophilic	[35]
FOYE (8PUN)	71°C	CD	Thermophilic	This work
RmER (5OCS)	n.a.	n.a.	Mesophilic	[37]

**FIGURE 5:**
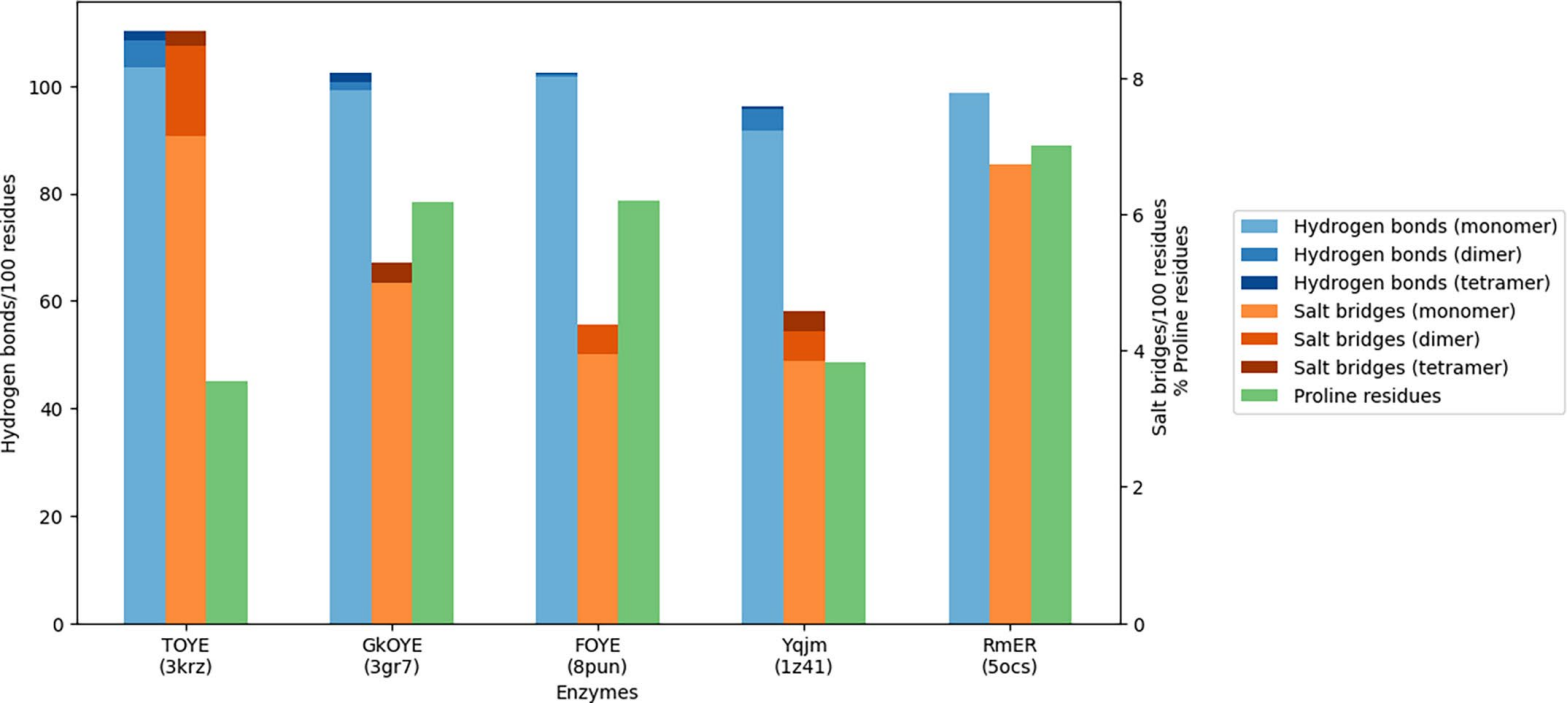
Hydrogen bonds, salt bridges, and proline count in the four tetrameric Old Yellow Enzymes (FOYE, Yqjm, GkOYE, and TOYE) and the closest crystallized homolog of FOYE (RmER).

Several parameters affect thermal stability, and their contribution varies from one protein family to another [38]. In OYEs, polar contacts and proline content have proven to be determining factors for thermal stability [39, 40, 41]. Thus, for our analysis, we calculated the intra‐ and intermolecular polar interactions using Hbplus software. Comparing FOYE with the other tetrameric OYEs reasonably explains the thermal behavior of this enzyme. FOYE displays a relative amount of hydrogen bonds comparable to GkOYE, definitely higher than the mesophilic Yqjm, but lower than the thermophilic TOYE. Curiously, FOYE appears to have the lowest number of salt bridges among the tetrameric OYEs, which justifies the lower melting temperature compared to GkOYE and TOYE. The relative amount of prolines is comparable to GkOYE and higher than TOYE and Yqjm, suggesting that a quite rigid structure in FOYE also contributes to thermal stability (Figure 5).

The closest homolog to FOYE is RmER, an enzyme from a mesophilic organism that displays a mesophilic temperature optimum [37]. RmER displays a lower amount of H‐bonds; however, at the same time, it possesses a higher amount of salt bridges and prolines. Despite the high sequence similarity with FOYE (55% identity, 71% similarity), RmER has been proven to be monomeric in solution [37]. The lack of a quaternary structure in this enzyme is due to its peculiar C‐terminal domain, which has a 14‐residue extension in the proximity of the arginine finger [17]. We speculate that such an extension might also cause instability of this enzyme at high temperatures.

## 4 Discussion

The structural and bioinformatic characterization of FOYE underlines its unique features as tetrameric Class II OYEs, revealing critical insights into the molecular basis of FOYE's tetramerization and thermal stability. FOYE's tetrameric structure is supported by a network of polar and hydrophobic interactions at the dimer–dimer interface, which we found to be spatially conserved among the other tetrameric OYEs. In any case, the distortion of helix 7 suggests that while the tetramerization mechanism is generally preserved across Class II OYEs, these enzymes can evolve unique structural adaptations to optimize the tetramer stability.

Despite the unusuality of finding a thermostable enzyme in a mesophilic organism, it is likely that the environment where *Ferrovum* sp. JA12 grows exerted evolutionary pressure toward these characteristics. Indeed, bioleaching reactions used by bacteria to extract electrons from metals in solution are strongly exothermic and can increase the temperature of the water up to 70°C [42]. Temperature tolerance must provide a clear evolutionary benefit in such an environment. Organisms living in mining‐associated habitats might be a good source for isolating thermotolerant enzymes.

FOYE's characteristics highlight the potential of mining‐associated microorganisms as a reservoir for thermotolerant enzymes. The high number of hydrogen bonds and proline residues in FOYE justify its thermal stability; however, the comparison with RmER parameters suggests that some other factors might contribute to the high melting temperature of our enzyme. A possibility is definitely the stabilization given by the reduced solvent exposure. The solvation‐free energy gain upon oligomerization (Δ*G* ^int^, Table S2) is indeed consinstent with this hypothesis.

## 5 Conclusions

In our research, we investigated the residues contributing to the formation of tetrameric quaternary structures in OYEs. Despite the unique aminoacidic variations and the structural distortion of helix 7 in FOYE, we found that the spatial location of the polar and hydrophobic interactions at the dimer–dimer interface is overall conserved in Class II OYEs. The formation of the tetramer introduces additional polar interactions while sequestering several hydrophobic residues from the solvent, contributing to the enhanced stability of this quaternary structure. Our work provides a substantial description of the spatial location and conservation of these interactions. This information can be used for the search or the design of tetrameric OYEs.

## Author Contributions

**Nakia Polidori:** conceptualization, investigation, writing – original draft, validation, supervision, formal analysis, software, methodology, visualization. **Peter Babin:** investigation. **Bastian Daniel:** funding acquisition, investigation, writing – review and editing, methodology, supervision. **Karl Gruber:** funding acquisition, writing – review and editing, visualization, software, supervision, project administration, conceptualization.

## Conflicts of Interest

The authors declare no conflicts of interest.

### Peer Review

The peer review history for this article is available at https://www.webofscience.com/api/gateway/wos/peer‐review/10.1002/prot.26800.

## Supporting information


**Data S1.** Supporting Information.

## Data Availability

The data that support the findings of this study are available from the corresponding author upon reasonable request.
