# Supplementary material for: Structure, Oligomerization, and Thermal Stability of a Recently Discovered Old Yellow Enzyme
Source: Proteins. 2025 Jan 22;93(6):1181–8. doi: 10.1002/prot.26800 (PMC12046209; doi:10.1002/prot.26800)
Supplement: Supplementary file 1 — Data S1. Supporting Information. [file PROT-93-1181-s001.docx]

**Supplementary material**

**DNA sequence of the FOYE construct:**

ctagaaataattttgtttaactttaagaaggagatataccaTGGGCAGCAGCCATCATCATCATCATCACAGCAGCG GCCTGGTGCCGCGCGGCAGCCATATGAGCCTGCTGTTTAGCCCGTATCAGCTGGGTAGCCTGAGCCT GGCAAATCGTCTGGTTATTGCACCGATGTGTCAGTATAGCGCAGTTGATGGTATTGCACAGGATTGG CATCTGATGCATCTGGGTCGTCTGGCAATTAGCGGTGCAGGTCTGGTGATTGTTGAAGCAACCGGT GTTAATCCGGAAGGTCGTATTACCCCGTTTTGTCTGGGTCTGTATAACGATGAACAAGAAGCAGCAC TGGGTCGTATTGTTGCATTTGCACGTGAATTTGGTCAGGCCAAAATGGCAATTCAGCTGGCACATGC AGGTCGTAAAGCAAGCACCCGTCGTCCGTGGGATCCGGGTAGTCCGTATAGTCCGGAAGAAGGTG GTTGGCAGACCTGGGCACCGAGCGCCATTAAATTCTATGAAGAAAGCCTGACACCGCATCCGATGA GCATTGAAGATCTGGAAACCGTTAAACAGGATTTTGTGAATAGCGCAATTCGTGCAGAACGTGCAG GTTTTAAAGCAATTGAACTGCATGGTGCACATGGCTATCTGATTCATCAGTTTCTGAGTCCGCTGAGC AATCAGCGTCAGGATCAGTATGGTGGTAGTCTGGAAAATCGTATGCGTTATCCGCTGGAAATTCTGA GCGCAGTTAAACATGCACTGAGTGCAGAAATGGTTGTTGGTATGCGTATTAGCGCAGTGGATTGGG CACCTGGTGGTCTGACCATTGAAGAAAGTATTACCTTTAGCCAAGAATGCGAAAAACGTGGTGCCG GTTTTATTCATGTTAGTACCGGTGGTCTGGTTGCACATCAGCAGATTCCGGTTGGTCCGGGTTATCA GGTTGAACATGCACAGGCAATTAAACAGAATGTGAATATTCCGACCATGGCCGTTGGTCTGATTACC CATAGCGCACAGGCAGAAACCATTCTGAAAAGCGAACAGGCCGATATGATTGCAATTGCCCGTGCA GCACTGAAAAATCCGCATTGGCCGTGGACCGCAGCGCTGGAACTGGGTGATAAACCGTTTGCACCG CCTCAGTATCAGCGTGCACGTTAACtcgagcaccaccaccaccaccactgagatccggctgctaacaaagcccgaaag gaagctgagttggctgctgccaccgctgagcaataactagcataaccccttggggcctctaaacgggtcttgaggggttttttgctga aaggaggaactatatccggat

**Protein sequence of the FOYE construct:**

MGSSHHHHHHSSGLVPRGSMSLLFSPYQLGSLSLANRLVIAPMCQYSAVDGIAQDWHLMHLGRLAISG AGLVIVEATGVNPEGRITPFCLGLYNDEQEAALGRIVAFAREFGQAKMAIQLAHAGRKASTRRPWDPGS PYSPEEGGWQTWAPSAIKFYEESLTPHPMSIEDLETVKQDFVNSAIRAERAGFKAIELHGAHGYLIHQFLS PLSNQRQDQYGGSLENRMRYPLEILSAVKHALSAEMVVGMRISAVDWAPGGLTIEESITFSQECEKRGA GFIHVSTGGLVAHQQIPVGPGYQVEHAQAIKQNVNIPTMAVGLITHSAQAETILKSEQADMIAIARAALK NPHWPWTAALELGDKPFAPPQYQRAR

| Wavelength (Å) | 0.97856 |
| --- | --- |
| Resolution range (Å) | 48.3 - 2.3 (2.382 - 2.3) |
| Space group | P 4_1_ 2 2 |
| Unit cell (Å, °) | 187.71 187.71 129.32 90 90 90 |
| Total reflections | 1318433 (135174) |
| Unique reflections | 102408 (10081) |
| Multiplicity | 12.9 (13.4) |
| Completeness (%) | 99.43 (99.78) |
| Mean I/sigma(I) | 17.09 (4.38) |
| Wilson B-factor (Å^2^) | 35.09 |
| R-merge | 0.1039 (0.6274) |
| R-meas | 0.1084 (0.6523) |
| R-pim | 0.03033 (0.1776) |
| CC1/2 | 0.998 (0.977) |
| CC* | 1 (0.994) |
| Reflections used in refinement | 101863 (10062) |
| Reflections used for R-free | 5135 (478) |
| R-work | 0.1806 (0.2570) |
| R-free | 0.2053 (0.2732) |
| CC(work) | 0.973 (0.938) |
| CC(free) | 0.964 (0.928) |
| Number of non-hydrogen atoms | 11933 |
| macromolecules | 11077 |
| ligands | 214 |
| solvent | 642 |
| Protein residues | 1424 |
| RMS(bonds) | 0.003 |
| RMS(angles) | 0.56 |
| Ramachandran favored (%) | 97.67 |
| Ramachandran allowed (%) | 2.33 |
| Ramachandran outliers (%) | 0.00 |
| Rotamer outliers (%) | 0.62 |
| Clashscore | 2.28 |
| Average B-factor (Å^2^) | 39.67 |
| macromolecules | 39.37 |
| ligands | 43.33 |
| solvent | 43.64 |

Table S1. Statistics of X-ray data collection and refinement of the crystal structures of FOYE (PDB: 8PUN). Statistics for the highest-resolution shell are shown in parentheses.


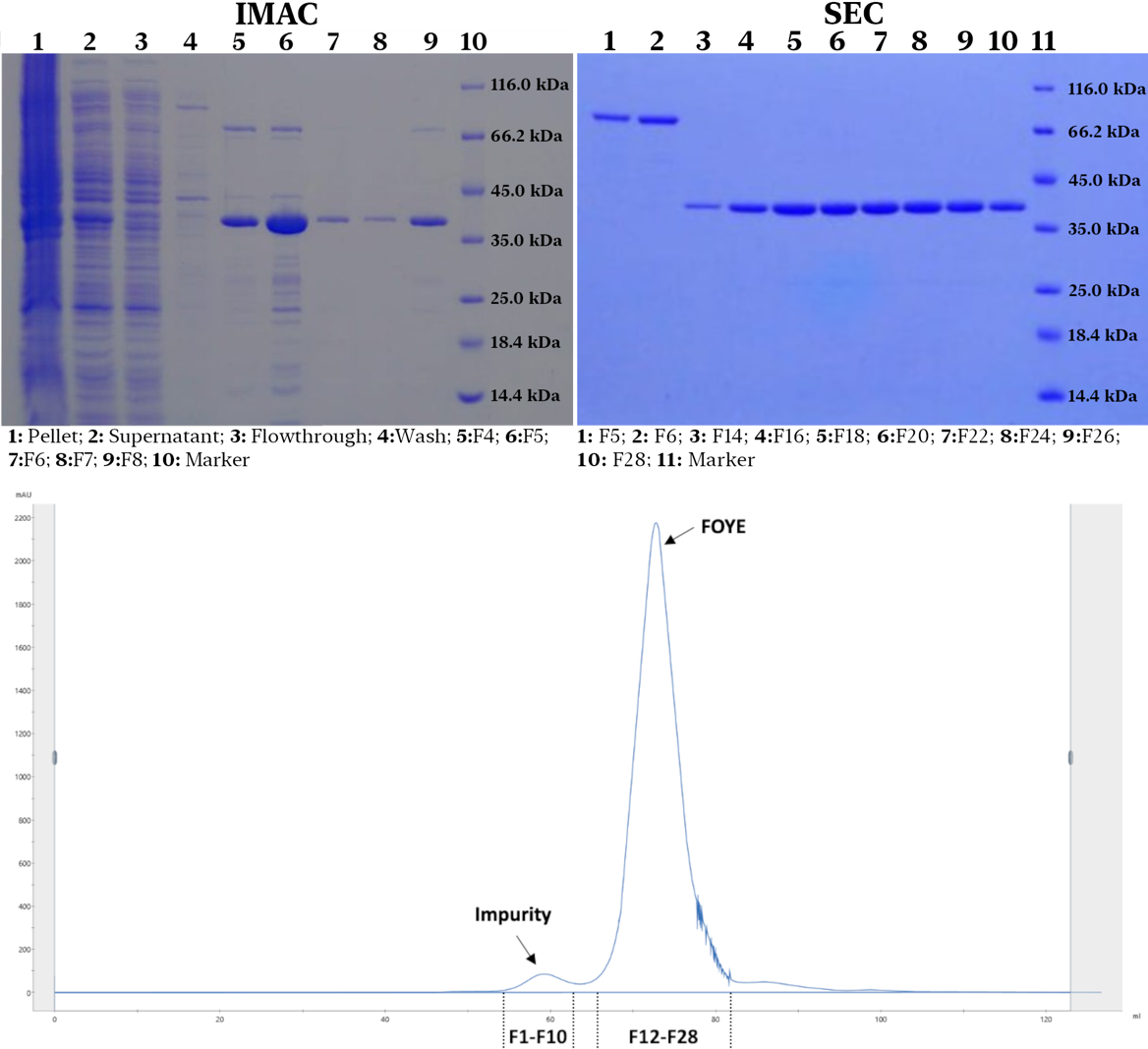


Figure S1. SDS-PAGE of the two chromatographic steps and the elution profile of the size-exclusion chromatography.


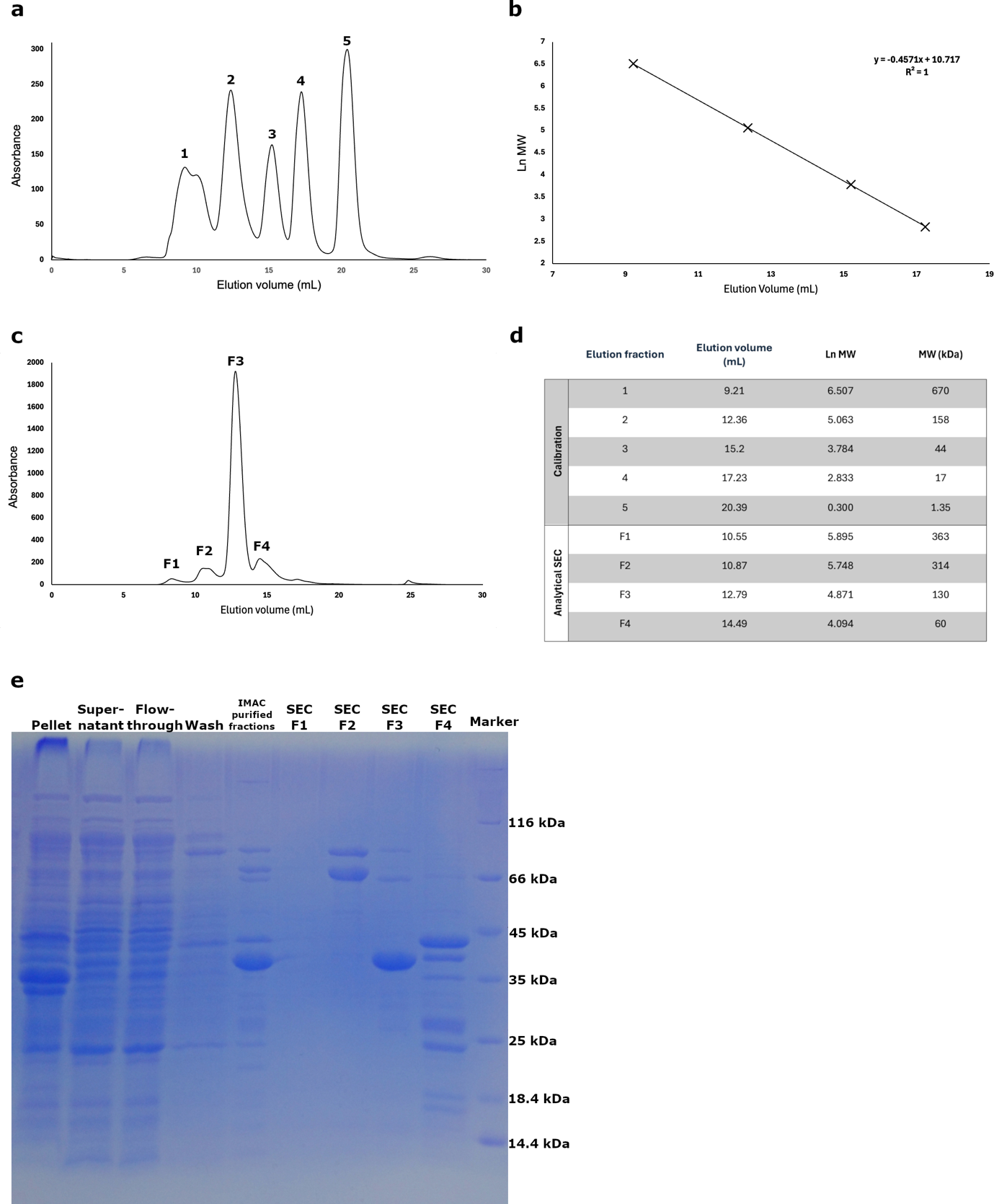


Figure S2 a) Chromatogram of the Biorad standard used for calibration of the elution volumes. b) Calibration curve built used the Biorad standard. MW: Molecular Weight c) Chromatogram of the analytical SEC performed on FOYE. d) Elution volumes of the calibration standards and the FOYE fractions. e) SDS-PAGE of the FOYE purification for the analytical size-exclusion chromatography.


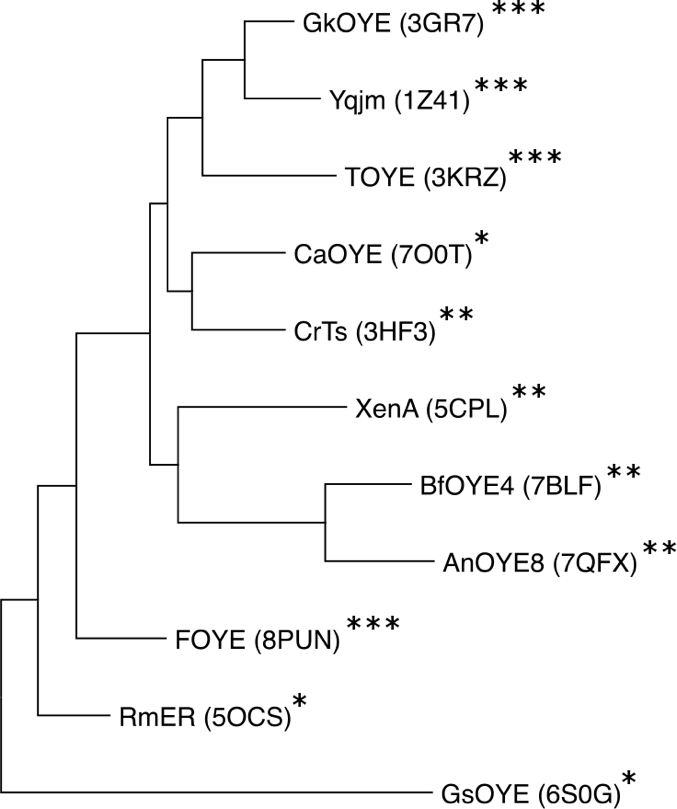


Figure S3. Phylogenetic tree of class II OYEs with a deposited structure. The class I enzyme GsOYE was used as an outlier. Stars indicate the oligomerization state in solution: * monomeric; ** dimeric; *** tetrameric.


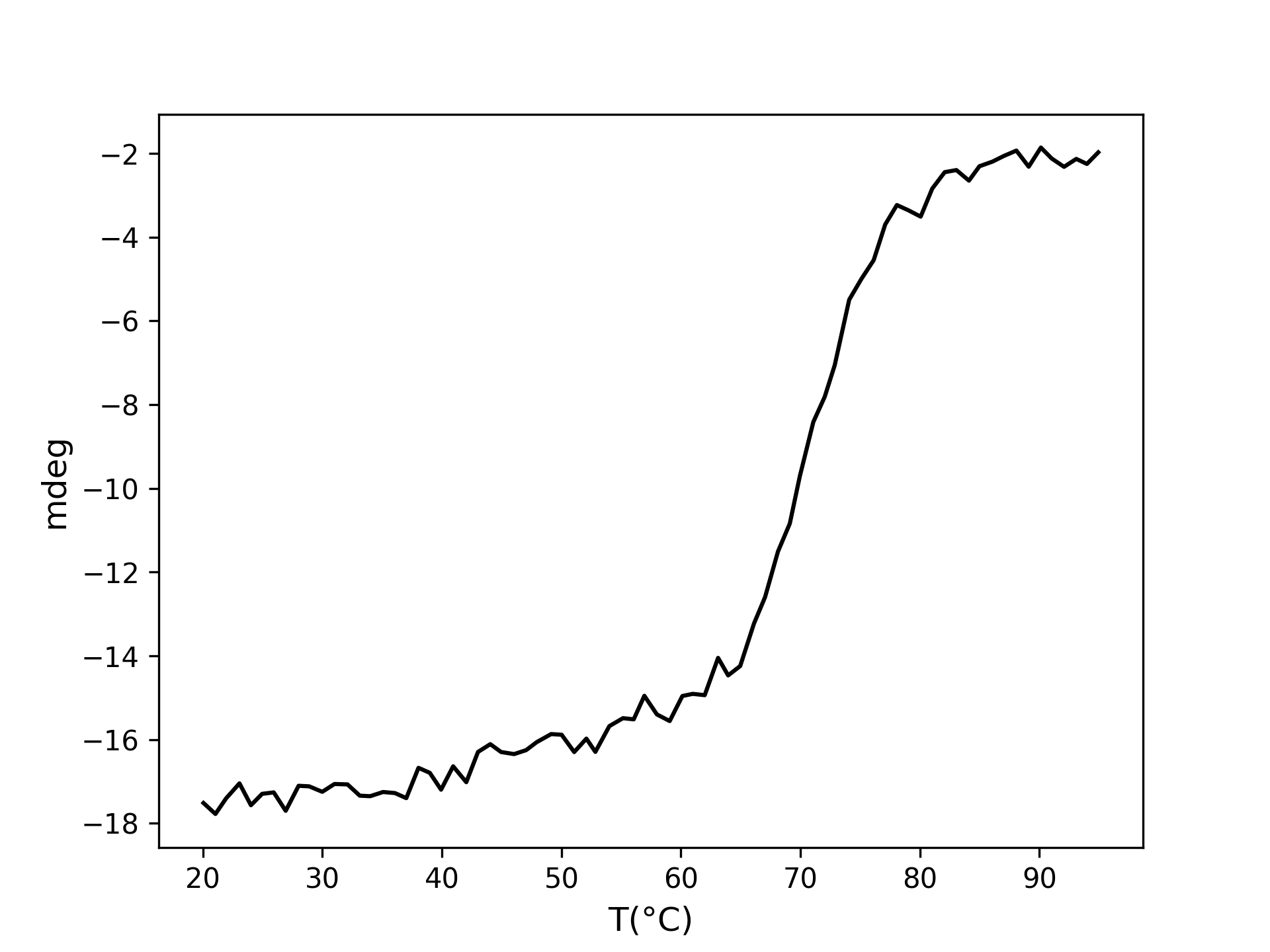


Figure S4. Melting temperature curve of FOYE measured trough CD spectroscopy.

|  | Assembly | Surface area (Å^2^) | Buried area (Å^2^) | ΔG^int^ (kcal/mol) | ΔG^diss^ (kcal/mol) |
| --- | --- | --- | --- | --- | --- |
| FOYE | Tetramer | 45660 | 17910 | -116.7 | 1.6 |
|  | Dimer 1 | 25160 | 6170 | -57.3 | 8.4 |
|  | Dimer 2 | 24820 | 7430 | -50.4 | 3 |
| GkOYE | Tetramer | 45300 | 13270 | -190.7 | 4.9 |
|  | Dimer 1 | 24120 | 5160 | -91.1 | 9.6 |
|  | Dimer 2 | 24040 | 5240 | -88.5 | 8.8 |
| TOYE | Tetramer | 45140 | 14130 | -31.7 | 2.1 |
|  | Dimer 1 | 24080 | 5530 | -12 | 10.5 |
|  | Dimer 2 | 24050 | 5600 | -13.4 | 9.1 |

Table S2 EBI-PISA assembly analysis of the tetrameric OYEs. ΔG_int_: solvation free energy gain upon formation of the assembly (value does not include the effect of satisfied hydrogen bonds and salt bridges across the assembly's interfaces). ΔG_diss_: free energy of assembly dissociation. Yqjm could not be analysed because it is predicted to be a dimer by the server.
